# Gains attained in malaria control coverage within settings earmarked for pre-elimination: malaria indicator and prevalence surveys 2012, Eritrea

**DOI:** 10.1186/s12936-015-0992-9

**Published:** 2015-11-20

**Authors:** Araia Berhane, Selam Mihreteab, Hagos Ahmed, Assefash Zehaie, Usman Abdulmumini, Emmanuel Chanda

**Affiliations:** Communicable Diseases and Control Division, Ministry of Health, Asmara, Eritrea; National Malaria Control Programme, Ministry of Health, Asmara, Eritrea; National Statistics Office, Asmara, Eritrea; WHO Eritrea Country Office, Asmara, Eritrea; Vector Control Specialist/Consultant, Lusaka, Zambia

**Keywords:** Malaria indicator and prevalence survey, Insecticide-treated nets, Indoor residual spraying, Access to treatment, Knowledge levels, Eritrea

## Abstract

**Background:**

Eritrea, like most countries in sub-Saharan Africa, has expended much effort towards malaria control with the view of transitioning from reduction of the disease burden to elimination. This paper reports on the level of achievement as highlighted by the follow-on, malaria-endemic area representative, survey that aimed to provide data and to assess progress on malaria indicators and parasite prevalence at household level across the country.

**Methods:**

In 2012, data were collected using a two-stage stratified cluster random sample of 1887 households in 96 clusters (villages in rural areas and census enumeration areas in urban centers) during a malaria indicator and prevalence survey in Eritrea. The survey determined parasite prevalence in vulnerable population groups and evaluated coverage, use and access to malaria control services. Standardized Roll-Back Malaria Monitoring and Evaluation Reference Group household and women’s questionnaires were adapted to the local situation and used for collection of data that were analysed and summarized using descriptive statistics.

**Results:**

The results of the survey showed that 90 % (95 % CI 89–91) of households owned at least one mosquito net. The proportion of the population with access to an insecticide-treated net (ITN) in their household was 55 % (95 % CI 54–56). The utilization of ITNs was 67 % (95 % CI 65–70) for children under 5 years and 60 % (95 % CI 58–63) for pregnant women (OR: 0. 73(95 % CI 0.62–0.85); P = 0.52). Only 28 % (95 % CI 26–30) of households were covered by indoor residual spraying (IRS) the previous year with significant heterogeneity by zoba (Debub 50 % (95 % CI 45–54) vs Gash Barka 32 % (95 % CI 28–36); OR = 0. 47 (95 % CI 0.36–0.61), P = 0.05). Malaria parasite prevalence was low; 1.1 % (95 % CI 0.9–1.3) in the general population and 1.4 % (95 % CI 1.0–2.0) in children under five and 0.7 % (95 % CI 0.4–1.1) among women aged 15–49 years. Only 19 % (95 % CI 15–26) of children under five had fever in the 2 weeks preceding the survey, with 61 % (95 % CI 54.1–67.1) seeking treatment from a health facility. Data on knowledge levels show that 92 % reported that malaria is transmitted by mosquitoes, 92 % mentioned that the use of mosquito nets could prevent malaria, 47 % knew malaria prevention medication, 83 % cited fever as a sign and symptom of malaria, and 35 % had heard or seen malaria awareness messages.

**Conclusion:**

Notwithstanding confounders, the observed low malaria parasite prevalence could be associated with malaria intervention coverage, access and utilization as well as high and equitable knowledge levels in the population. This indicates that Eritrea is on the right track towards pre-elimination. However, technical and infrastructure capacity should be strengthened to facilitate implementation, surveillance, monitoring, and evaluation.

## Background

Malaria continues to be a vector-borne disease of public health significance worldwide. This is reflected in the high figures of morbidity, mortality and transmission intensity associated with the disease [1]. The situation in malaria-endemic countries is particularly grave among children and pregnant women [2, 3]. Considerable efforts have been expended towards the control and elimination of the disease by Roll Back Malaria (RBM) partners and stakeholders but with varied levels of success [1]. Since the malaria eradication era through 2012, only 30 countries have been certified to have eliminated the disease [4]. The recently launched Global Technical Strategy for Malaria (2016–2030) supports countries in reducing the disease burden and accelerating progress towards elimination [5]. While several documents to guide the transition from malaria control to elimination exist [6, 7], endemic countries are grappling with a diversity of constraints as they strive towards the attainment of the malaria elimination goal [8].

Eritrea lies north of the Equator between latitudes 12°22′ and 18°02′N, and longitudes 36°26′21″E and 43°13′E in the Horn of Africa. It covers an area of 124,000 sq km and a population of 3.6 million with higher density in highlands than in lowlands [9]. Altitude ranges from 0 to >3000 m above sea level. Temperatures range from 16 to 45 °C with the lower lands being hotter and drier. Malaria is endemic in four of the six administrative zobas (regions): Anseba, Debub, Gash-Barka, and Semenawi Keih Bahri. The disease is one of the major public health problems in Eritrea with more than 60 % of the burden occurring in Gash Barka. Currently, the construction of mini-dams, introduction of irrigation schemes, traditional mining projects, and movement of non-immune people to these areas may worsen the malaria situation. About 70 % of the population resides in malaria-endemic areas [9]. *Anopheles arabiensis* is the primary malaria vector, a species difficult to control using conventional interventions due to its facultative indoor and/or outdoor feeding and resting behaviour. *Anopheles d’thali, Anopheles cinereus, Anopheles rhodesiensis, Anopheles squamosus*, and *Anopheles rupicolus* are secondary vectors [9]. Malaria peaks in October in most zobas, while March–April is the main transmission season in the coastal area.

The country has implemented very successful malaria control since the establishment of the National Malaria Control Programme (NMCP) in 1995 [10]. The World Health Organization (WHO)-recommended case management and vector control tools have been implemented extensively [10]. In the context of integrated vector management (IVM), the vector control programme combines high coverage of long-lasting insecticidal nets (LLINs), selected indoor residual spraying (IRS) and targeted larval source management (LSM). Case management involves early definitive diagnosis by microscopy or rapid diagnostic tests (RDTs), prompt and effective treatment of uncomplicated malaria using artesunate-amodiaquine combination therapy (ACT), and quinine is used for severe/complicated cases and malaria in pregnancy, and intermittent presumptive treatment (IPT) using at least two doses of sulfadoxine/pyrimethamine (SP) given to pregnant mothers. These efforts have been supported by information, education and behavioural change communication (IEC/BCC) [9, 10]. As a result, Eritrea has made exceptional progress in malaria control efforts in the past decade with available data indicating that the disease burden is decreasing from year to year [11, 12]. Presently, Eritrea is one of the countries in sub-Saharan Africa with great potential to accelerate progress from malaria control towards elimination. The country envisions achieving this through a phased approach with some zobas targeting identified foci to interrupt transmission and others consolidating control before entering the pre-elimination phase [13].

Eritrea, like several other malaria-endemic countries, has endeavoured to measure the coverage and impact of malaria control tools [1]. Malaria-endemic area representative malaria indicators surveys (MIS) were conducted in 2004 and 2008 with the support of the Government and partners [11] based on the guidelines developed by the RBM Monitoring and Evaluation Reference Group (MERG) [14]. These surveys had focused on knowledge and coverage of interventions. In October 2008 the coverage of at least one LLIN per household was 33 %. Usage of LLINs was 49 and 44 % among children under 5 years old and in women aged 14–49 years, respectively. Twenty-three per cent of households had been sprayed in the previous 6 months, and 53 % of children with fever took an anti-malarial drug [11]. In 2012, the country conducted a follow-up Malaria Indicator and Prevalence Survey that included biomarkers to measure the burden of parasitaemia in various age groups to understand better the magnitude and patterns of malaria infection within the general population, particularly in children under five and women in reproductive-age population [11]. The main objective of the survey was to measure progress towards achieving the goals and targets set in the Malaria Strategic Plan 2010–2014 [10]. This study reports on the findings of the survey and the progress that Eritrea has made towards national and global targets on the long path towards malaria elimination in the country.

## Methods

### Study design and sampling approach

The Malaria Indicator and Prevalence Survey was conducted between September and October 2012 in accordance with the RBM MERG protocol [11, 14] adapted to local settings. The survey utilized a two-stage, stratified, cluster, sampling frame designed to provide malaria-endemic areas representative estimates of key malaria indicators. The sampling frame comprised the list of villages in rural areas and census enumeration areas (EAs) in urban areas with their respective number of households. The sample was stratified into four survey zones: Anseba, Debub, Gash-Barka, and Semenawi Keih Bahri. First, an overall sample of 96 clusters (EAs) was selected as primary sampling units and stratified by urban/rural; 23 in urban areas and 73 in rural areas, with probability proportional to size and a complete listing of all households in each cluster was carried out. The sample allocation among the zobas and urban–rural areas was not proportional and therefore not self-weighting in the four strata. The weights were calculated based on the sampling frame. Second, 42 households per EA were selected for interviewing using equal probability systematic random sampling, making a total sample of 1,887 households. In case of absence of all eligible respondents or subjects, up to three visits were made to ascertain compliance and to minimize potential bias [11].

### Survey questionnaires

The survey used standard MIS questionnaires based on the RBM MERG guidelines with modification to reflect relevant issues of malaria in Eritrea [14, 15]. The household questionnaire was used to list all the usual members and visitors in the selected households and to collect basic information on the characteristics of each person listed, including age, sex, household’s residence and assets, and ownership, type and use of mosquito nets. The data on the age and sex of household members obtained in the household questionnaire were used to identify eligible women for individual interview and children aged 0–59 months for malaria testing. The women’s questionnaire was used to collect information from all women aged 15–49 years on background characteristics, full reproductive history, prenatal care and preventive malaria treatment for most recent birth, prevalence and treatment of fever among children under 5 years, including knowledge about malaria causes, symptoms, and danger signs of malaria prevention and treatment, antenatal care (ANC) service utilization [11].

### Survey organization, training and data collection

The survey designing, planning and implementation was a collaborative effort of multiple local and international malaria stakeholders. A Five-day training activity was conducted in August, 2012 for 100 field staff: 32 interviewers, 16 supervisors, four zoba supervisors, and two national-level survey coordinators, 32 laboratory technicians, eight laboratory supervisors, and six laboratory coordinators. The training was provided by experts from National Statistics Office (NSO), NMCP, WHO, and Economic and Social Consulting (ECOSOC) based on the RBM MERG interviewer and supervisor manuals. Additionally, 40 medical laboratory technologists were trained on malaria testing based on national guidelines. The team supervisors and editors were further trained in data quality control procedures and fieldwork coordination. Prior to fieldwork the questionnaires were pre-tested on a random sample of households in Hibmirti. Information about the MIS was dispatched to the communities residing in the sampled clusters through the respective administrative channels [11]. Sixteen teams, each comprising two interviewers, two laboratory technicians and one supervisor were responsible for data collection for 20 days between September and October 2012.

### Malaria diagnosis and treatment

The biomarkers in the survey included RDTs and blood slides for microscopic examination for malaria. Blood samples were collected from a finger prick using a single-use, spring-loaded, sterile lancet. All the tests were performed simultaneously from a single finger prick. *Plasmodium falciparum* malaria testing was done using the Paracheck Pf ™ RDT, which has shown good sensitivity and specificity in operational settings [16]. Test results for RDTs were provided to a child’s parent/guardian verbally and were recorded on the household questionnaire. Two blood slides, thick and thin films, were prepared for each participant by a laboratory technologist as per standard WHO-approved protocol [17]. The blood slides were air-dried, fixed (thin films), stained with Giemsa and transported to the reference National Health Laboratory (NHL) in Asmara for reading. Based on standard laboratory malaria microscopy procedures, the microscopists determined the presence, density (thick blood film) and species of the malaria parasites (thin blood film). If no parasites were found after examination of 200 high power fields, the thick blood smear was considered negative and the corresponding thin blood film was not read. Quality control was done by cross-checking all positive blood slides plus 10 % of the negative slides. Malaria prevalence was determined by microscopy. Children who tested positive for malaria using the RDT were offered a full course of treatment according to the standard protocol for treating malaria in Eritrea [18]. All severe cases with a positive RDT result were referred to a health facility for follow-up evaluation and treatment.

### Data management and analysis

Data were entered using the CS Pro version 4. 0 (Census and Survey Processing System) software package. All completed questionnaires were double entered, achieving 100 % verification to eliminate key-in error during entry. Descriptive statistics were used to illustrate the characteristics of the sample and calculate coverage, use and access estimates. The Statistical Package for Social Sciences (SPSS version 18. 0; Inc, Chicago, IL, USA) was used to analyse the data. A proportion or odds and the comparison of two proportions or odds were computed to obtain confidence intervals and odds ratios. P-values were computed by using the Chi square statistic. All analyses were based on weighted data initially calculated based on the sampling frame and later adjusted to cater for the household and individual interview non-responses.

### Quality control

To ensure high quality data collection, the teams were supervised and monitored daily. All team supervisors were experts from the National Statistics Office (NSO), Ministry of Health (MOH), NHL, zoba referral hospitals and health centres. The teams randomly inspected completed households to confirm correctness of records obtained from the survey and completion of supervisory checklist, and observed a team’s overall performance as well as providing feedback and sharing the experiences of other teams. The quality of data entering and analysis was checked by highly qualified statisticians.

### Ethical clearance

The survey protocol received ethical clearance from the MOH research division ethical committee. Written or verbal informed consent was obtained from the heads of households and each eligible individual before conducting the household questionnaires. Additional in-formed consent from a child’s parent or guardian and the pregnant women for blood films and anti-malarial treatment with ACT was provided by a nurse or physician when participants had a positive RDT result.

## Results

### Attributes of sampled population

This survey was conducted using a malaria-endemic area representative sample of 1887 households in 96 EAs. The response rates were: 96 % (95 % CI 95–97), (n = 1818) for households; 0 % (95 % CI 88–91), (n = 1895) for women interviewed; 73 % (95 % CI 73–74), (n = 16,292) for malaria prevalence. The data indicate that 8533 people were enumerated in the survey with males constituting 47 % (95 % CI 46–48) and females 53 % (95 % CI 52–54) of the population.

### Net ownership at household level

Overall, 90 % (95 % CI 89–92) of households had at least one mosquito net, 88 % (95 % CI 87–90) had at least one ever-treated bed net, 87 % (95 % CI 85–88) had at least one insecticide-treated net (ITN), and 86 % (95 % CI 84–87) had at least one LLIN (Table 1). Overall, coverage of at least one LLIN per household increased from 33 % in 2008 to 86 %, representing 158 % increase. There was no significant difference in ownership of nets between urban areas 85 % (95 % CI 82–88) and rural areas 87 % (95 % CI 85–89); OR = 1.15 (95 % CI 0.84–1.56), P = 0. 90. There was great variability by zoba (Anseba 92 % (95 % CI 89–94) vs Semenawi Keih Bahri 56 % (95 % CI 52–61); OR = 0. 11 (95 % CI 0.08–0.17), P = 0.001). The survey also showed that 62 % of households had more than one ITN and 61 % had more than one LLIN.

**Table 1:** Net ownership by households with at least one mosquito bed net

	Number of households	Having bed net	Having ever-treated bed net	Having ITN	Having LLIN
	N	% (95 % CI)	% (95 % CI)	% (95 % CI)	% (95 % CI)
Residency					
Rural	436	90 % (87–93)	89 % (85–91)	85 % (82–88)	85 % (81–88)
Urban	1382	90 % (88–92)	88 % (86–90)	87 % (85–89)	86 % (84–88)
Zoba					
Anseba	472	95 % (92–96)	93 % (90–95)	92 % (0–94)	92 % (89–94)
Debub	471	93 % (90–95)	92 % (89–94)	90 %(87–92)	88 % (85–91)
Gash Barka	463	93 % (90–95)	91 % (88–93)	88 % (85–91)	88 % (85–91)
Semenawi KeihBahri	412	64 % (59–68)	59 % (55–64)	57 % (52–62)	56 % (52–61)
Total	1818	90 % (87–91)	88 % (87–90)	87 % (85–88)	86 % (84–87)

*N* number, *%* frequency

### Access and utilization of nets

Overall the proportion of the population with access to an ITN in their household was 55 % (95 % CI 54–56). It varied markedly by zoba (Table 2), being highest in Gash Barka 66 % (95 % CI 64–68) and lowest in Semenawi Keih Bahri 21 % (95 % CI 18–23); OR = 0.14 (95 % CI 0.11–0.16), P < 0.0001). Generally, 67 % (95 % CI 65–70) of children under five compared to 60 % (95 % CI 58–63) of pregnant women slept under an ITN the night preceding the survey (OR: 0.73 (95 % CI 0.62–0.85); P = 0.52). Usage of LLINs increased from 49 % in 2008 to 67 % in 2012 among children under 5 years old and from 44 % in 2008 to 60 % in 2012 in women aged 14–49 years, representing 38 and 36 % increase, respectively. Equally, variability was observed in mosquito net usage by zoba (Table 2). The likelihood of children sleeping under a net was highest in Gash Barka 77 % (95 % CI 73–82) and lowest in Semenawi Keih Bahri 32 % (95 % CI 26–39); *P* for variation <0.0001. Pregnant women in Semenawi Keih Bahri 25 % (95 % CI 20–31) were less likely to sleep under a mosquito net than those in Gash Barka 72 % (95 % CI 68–76); P < 0. 0001).

**Table 2:** Access to and use of ITN

	Population (de-facto)	People with access to ITN within the household	People who slept under an ITN the previous night	
			Children under 5 years	Women aged 15–49 years
	N	% (95 % CI)	% (95 % CI)	% (95 % CI)
Residency				
Rural	1750	55 % (53–57)	68 % (65–71)	60 % (57–63)
Urban	5332	56 % (54–57)	66 % (60–71)	60 % (55–65)
Zoba				
Anseba	2092	66 % (64–68)	74 % (70–79)	71 % (67–75)
Debub	2024	49 % (46–51)	64 % (58–69)	53 % (48–58)
Gash Barka	1924	66 % (64–68)	77 % (73–82)	72 % (68–76)
Semenawi Keih Bahri	1042	21 % (18–23)	32 % (26–39)	25 % (20–31)
Total	7082	55 % (54–56)	67 % (65–70)	60 % (58–63)

*N* number, *%* frequency

### Indoor residual spraying

Only 28 % of 1818 surveyed households had been sprayed in the previous 6 months. Among households sprayed in the previous 6 months, 88 % (95 % CI 87–90) also had at least one ITN (Table 3). Heterogeneity was observed in the number of sprayed households by zoba (Debub 50 % (95 % CI 45–54) vs Gash Barka 32 % (95 % CI 26–36); OR = 0. 47 (95 % CI 0.36–0.61), P = 0.05) but no difference was observed for urban 26 % (95 % CI 22–30) vs rural 29 % (95 % CI 27–31); OR = 1.18 (95 % CI 0.93–1.51), P = 0.65). Overall, the percentage of households living in a sprayed dwelling has increased slightly from 23 % in 2008 to 28 % in 2012, an increase of 25 %. The increase is substantially higher among those households in the highest wealth quintile (from only 3.8 to 20 %), in urban areas (10–25 %) and in zoba Debub (27–49 %).

**Table 3:** Households covered by IRS and those having an ITN

	Households covered by IRS		Household also having ITN
	N (%)	(95 % CI)	% (95 % CI)
Residency			
Rural	1382 (29)	27–31	89 % (87–90)
Urban	436 (25)	22–30	87 % (83–90)
Zoba			
Anseba	472 (1. 0)	0.99–1.00	93 % (90–95)
Debub	471 (49)	45–54	93 % (90–95)
Gash Barka	463 (32)	28–36	0 % (87–92)
Semenawi Keih Bahri	412 (2. 0)	16–24	59 % (54–63)
Total	1818 (28)	26–30	88 % (87–90)

*N* number, *%* frequency

### Level of malaria knowledge

Of the women interviewed, 92 % (95 % CI 90–93), n = 1696 reported that malaria is transmitted by mosquito bites; 85 % (95 % CI 83–86) sited fever as a sign and symptom of malaria, 58 % feeling cold, 49 % nausea and vomiting, 48 % headache, 47 % body ache or joint pain, and 19 % loss of appetite. Respondents cited sleeping under a mosquito net, 92 % (95 % CI 90–93) and taking preventative medicine, 47 % (95 % CI 45–49), as methods for avoiding malaria (Table 4). Respondents also reported on socio-economic impact of malaria: absenteeism from work 25 % (95 % CI 22–27) and school 9 % (95 % CI 8–11). The main sources of information on malaria were health workers or health facilities (36 %), community meetings (36 %), radio (25 %), television (14 %), and community agents (24 %). The interviewees reported having heard/seen messages about: early seeking of treatment (43 %), environmental management (64 %) and ITN use (69 %).

**Table 4:** Respondents’ knowledge about transmission, prevention and signs and symptoms

Respondents’ awareness	Residence		Zoba				Total % (95 % CI)
	Rural % (95 % CI)	Urban % (95 % CI)	Anseba % (95 % CI)	Debub % (95 % CI)	Gash Barka % (95 % CI)	Semenawi Keih Bahri % (95 % CI)	
Malaria is transmitted by a mosquito bite	91 % (0–93)	93 % (0–95)	96 % (94–97)	88 % (84–91)	93 % (90–95)	92 % (89–94)	92 % (90–93)
Sleeping under a mosquito net to prevent malaria	91 % (89–92)	93 % (90–95)	95 % (93–97)	91 % (88–93)	92 % (89–94)	86 % (83–89)	92 % (90–93)
Malaria prevention medication	44 % (42–47)	56 % (51–61)	44 % (39–48)	52 % (48–55)	43 % (39–47)	52 % (45–59)	47 % (45–49)
Fever as a sign and symptom of malaria	83 % (80–84)	85 % (81–88)	88 % (84–90)	77 % (72–80)	86 % (82–88)	87 % (84–90)	83 % (81–85)
Heard or seen malaria awareness messages	31 % (27–36)	46 % (39–53)	45 % (39–52)	39 % (32–46)	26 % (19–34)	29 % (22–37)	35 % (31–39)

*N* number, *%* frequency

### Malaria parasite prevalence

Overall, *P. falciparum* prevalence of malaria among general population was 1.1 % (95 % CI 0.9–1.3) (Table 5). Prevalence of infection was 1.4 % (95 % CI 1.0–2.0) in children under five and 0.7 % (95 % CI 0.5–1.1) among women age 15–49 years. Overall parasite prevalence ranged from 2. 6 % (95 % CI 2.1–3.2) in Gash Barka to zero in Anseba. The trend in children under five followed a similar pattern with 2.5 % (95 % CI 1.5–4.1) in Gash Barka and zero in Anseba. However, prevalence in women 15–49 years was higher in Debub 2.1 % (95 % CI 1.3–3.3) and lowest in Semenawi Keih Bahri 0.1 % (95 % CI 0.01–0.72).

**Table 5:** Parasite prevalence and access to anti-malaria treatment

	Malaria parasite prevalence			Access to anti-malaria treatment Children under 5 years		
	General population % (95 % CI)	Children under 5 years % (95 % CI)	Women aged 15–49 years % (95 % CI)	Had fever last 2 weeks % (95 % CI)	Sought treatment/advice % (95 % CI)	AS-AQ same day/next day % (95 % CI)
Residency						
Rural	1.3 % (1.0–1.6)	1.5 % (1.0–2.2)	1.9 % (1.4–2.5)	20 % (14–26)	56 % (52–67)	2.6 % (1.1–6.4)
Urban	0.5 % (0.3–0.8)	0.9 % (0.4–2.3)	0.1 % (0.02–0.64)	19 % (10–31)	65 % (52–77)	0
Zoba						
Anseba	0	0	0.2 % (0.1–0.8)	11 % (4–24)	60 % (46–75)	0
Debub	0.8 % (0.5–1.2)	1.5 % (0.8–3.0)	2.1 % (1.3–3.3)	21 % (13–33)	58 % (46–70)	1.7 % (0.3–8.8)
Gash Barka	2.6 % (2.1–3.2)	2.5 % (1.5–4.1)	1.9 % (1.5–3.8)	27 % (18–38)	63 % (52–73)	3.2 % (1.0–9.8)
Semenawi Keih Bahri	0.1 % (0.02–0.31)	0.2 % (0.03–1.38)	0.1 % (0.01–0.72)	11 % (4–25)	64 % (46–77)	0
Total	1.1 % (1.0–1.3)	1.4 % (1.0–2.0)	1.4 % (1.0–1.9)	19 % (15–26)	61 % (54–67)	2 % (0.8–4.9)

*N* number, *%* frequency

### Community access to anti-malaria treatment

Only 19 % (95 % CI 15–26) of children under 5 years had a fever in the 2 weeks preceding the survey (Table 5). This ranged from 11 % (95 % CI 4–25) in Semenawi Keih Bahri to 27 % (95 % CI 18–38) in Gash Barka zone. Advice and/or treatment sought from a health facility or healthcare provider increased from 53 to 61 % (95 % CI 54–67) of the children, representing a 10 % increase. Only 2 % (95 % CI 1–5) reported taking anti-malarial drugs on the same/next day (Table 5).

## Discussion

**Fig. 1:**
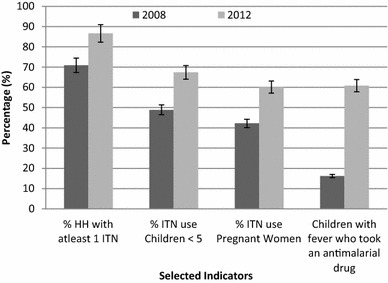
Progresss in key malaria programme indicators, 2008–2012

Global malaria control efforts are hinged on key strategies, which include prompt and effective case management, IPT and IVM. Malaria-endemic countries across sub-Saharan Africa are scaling up control efforts with a view to move towards elimination [6, 7, 13]. This malaria-endemic area-specific survey has demonstrated progress towards the RBM Abuja targets on coverage of interventions relative to household surveys conducted previously in Eritrea [11]. Appreciable progress in key malaria programme indicators has been observed between 2008 and 2012 (Fig. 1). This information is useful to the stakeholders for implementing decisions on malaria programming in the country. The observed levels of access to treatment (Table 5) are also consistent with what has been reported before by other countries in the region [19–21]. While early treatment-seeking behaviour and use of ITNs still fall well below the national and international targets of at least 80 %, enhancing IEC/BCC, addressing intra-household dynamics, mobility of populations, gender issues, and training of more health workers, including those at community level, will be critical in improving access and utilization. Currently the malaria programme is primarily funded by global funds and technically supported by the WHO. This entails the need for sustained adequate local financial resource mobilization and political commitment.

In Eritrea, some high altitude areas are free from malaria and control efforts have reduced the transmission intensity to low levels, allowing programme re-orientation towards pre-elimination in localized geographical areas. While the country has recorded exponential increases in some key indicators, sporadic malaria epidemics have been observed, mostly precipitated by mobility of populations and seasonal increase of malaria. The propensity for complacency with what has been achieved could be one of the other factors for increasing malaria [22]. However, considering the current malaria burden, can the prevailing level of indicators enable the country to achieve malaria elimination against the backdrop of geographical variation in implementation of interventions? Immediate steps need to be taken to ameliorate the situation. This will require pragmatic and strategic solutions to overcome the hindrance for achieving the set goals on time.

Due to their proven efficacy and operational ease of deployment, LLINs are the most implemented intervention for malaria vector control in malaria-endemic areas [23, 24]. Most countries have made appreciable progress in scaling up distribution and increasing utilization [9]. Generally, ownership of LLINs has been reported to be relatively higher than their utilization which still remains relatively low [19, 20]. The average number of LLINs per household improved from 0.5 in 2008 to 1.8 in 2012 and the percentage of households owning at least one ITN increased from 73 % in 2004 to 87 % in 2012, surpassing the global targets. The percentage of households owning more than one ITN increased from 40 % in 2008 to 62 % in 2012 (P = 0.029) [11]. However, net utilization the night before the survey remains much lower (Tables 1, 2) [25] with no significant difference between pregnant women and children under 5 years (P = 0.518). The levels of households having more than one ITN and proportion of population with access to bed nets in the household necessitates expending more effort towards universal coverage in Eritrea. Universal coverage of all households with adequate numbers of mosquito nets is critical to increase access and utilization, particularly by the two population groups. This would require investment in extensive IEC/BCC campaigns to raise the awareness of communities on the importance of using ITNs for malaria prevention. Unlike the experiences in other surveys where net ownership and use are higher in rural compared to urban areas [20], stratification by residence in this study did not exhibit any significant difference between urban and rural areas (Table 4). This could be ascribed to equitable access to pertinent key IEC/BCC messages in the two areas.

The RBM partnership advocates IRS as a key malaria vector control tool along with LLINs [25, 26]. Many malaria-endemic countries, including Eritrea, have adopted and implemented the intervention in the context of IVM with concomitant marked reduction in the disease burden [14, 27, 28]. Like many other countries, the implementation of IRS in Eritrea is minimal (Table 3). Data from this study indicate that only 28 % of the surveyed households had been sprayed in the previous 6 months, exhibiting great heterogeneity by zoba (Table 3). This is exemplified by the notable variation in coverage of IRS between zobas: Debub and Gash Barka (P = 0.046). The low coverage by intradomiciliary spraying 6 months preceding the survey is consistent with findings from Djibouti [21], Angola [29], Ethiopia [19], and Zambia [30]. This is largely because IRS is highly technical and logistically more difficult to deploy compared to ITNs. As such, only a few eligible areas are covered by the intervention in most endemic areas. Although the malaria risk is comparatively lower in urban areas than in rural areas, there is no significant difference in coverage of both IRS and LLINs by residence in Eritrea. Clearly, the country has generally made great progress in controlling malaria but still lacks definitive data to support and guide evidence-based deployment of effective interventions.

Since 2008, there have been changes in the distribution of interventions. In Gash Barka and Debub, IRS and LLINs have been deployed as front-line interventions supplemented by LSM with larviciding. However, IRS operations have, over the years, been conducted outside expert knowledge or skill. In Anseba and Semenawi Keih Bahri, on the contrary, distribution of LLINs is the only key intervention deployed on an operational scale supplemented by LSM. In Anseba Zone, while IRS was dropped for the reason of reduction in malaria cases, Hagaz and Kerhebet sub-zones persistently record high malaria cases. Convincingly, Semenawi Keih Bahri is characterized by nomadic populations and housing structures that are not amenable for IRS. The prevailing circumstances necessitate the generation of entomological and epidemiological evidence to guide selection of appropriate interventions. In Eritrea malaria vector control is driven by the public sector with very minimal engagement of the private sector.

The Global Malaria Programme has introduced a pre-elimination phase in the malaria programme continuum as a transition phase to re-orient programmes from control to elimination and subsequently to prevention of re-introduction [31]. Population-based surveys conducted in different sub-Saharan African countries have consistently reported high levels of parasite prevalence [19, 29, 30]. In Eritrea, the low malaria transmission and very low overall parasite prevalence rate prompted the malaria programme in 2013 [32]. This starting exercise classified the Eritrean malaria control programme as pre-elimination for satisfying the criteria namely: low parasite prevalence of 1.9 %, incidence of four per 1000, low number of deaths, ongoing elimination of *P. falciparum*, and limitation of malaria to certain villages in some sub-zones. The only unsatisfied criteria was high test positivity rate (TPR) of 19 % compared to <10 and >5 % recommended for consolidation, which was the result of clinicians testing suspected malaria cases rather than all fever cases. Unlike in other malaria-endemic settings, there was no significant difference in prevalence between rural and urban areas in both children (P = 0.699) and pregnant women (P = 0.203) in Eritrea (Table 5). Overall, knowledge levels regarding causes, preventive measures and treatment of malaria, LSM and community involvement are quite high. This could explain the corresponding increase in the use of malaria control tools observed in the country. Contrary to the findings of other surveys in sub-Saharan African countries [25–27], the high levels of knowledge on malaria control and prevention in this study mirror those for Zambia [30].

The goal of the elimination phase is to reduce the malaria burden to an incidence rate of less than one per 1000 people at risk at a sustainable level [33]. To achieve this, Eritrea needs to improve the surveillance response system and targeting of case management and vector control operations, in residual and new active foci. Establishment of a notification system and regulation of the limited private sector, which includes rural drug vendors, will be critical in this phase. Subsequently, the programme should aim to halt local malaria transmission through targeted interventions to populations at risk in malaria foci [33]. As entomology is a guide for effective intervention, requisite human resource, technical and logistical capacity will be necessary for timely and effective intervention and supervision at zonal and sub-zonal level. However, there is still geographical variation in the deployment of interventions to warrant an easy transition of the country from control to pre-elimination. This could probably be as a result of reducing resources due to falling infection rates.

The present findings validate the need for consistency in conducting population-based health surveys to generate empirical evidence for decision-making. Nevertheless, the study did not assess anaemia prevalence in children under five and pregnant women, despite its strong correlation with malaria infection, as it was not a prioritized indicator. Furthermore, this study could not consider analysis of the two additional RBM indicators of ‘proportion of households with at least one ITN for every two people’ and ‘proportion of population with access to an ITN within the household’ as recommended by MERG, together with the inherent gaps, i.e. households with no ITN, households with any but not enough ITN, and population with access to ITN not using it [30], due to lack of adequate data. To provide timely, accurate, sub-national- and district-level burden estimates throughout the year, a rolling MIS that adopts the standard cross-sectional evaluation tool into continuous monitoring could be useful [34].

## Conclusions

Notwithstanding confounders, the observed low malaria parasite prevalence could be associated with malaria intervention coverage and utilization indicators as well as high coverage and equitable knowledge levels. This indicates that Eritrea is on the right track towards pre-elimination of malaria. However, access, coverage and utilization of malaria control tools should be increased through evidence-based scaling-up interventions and improving IEC/BCC. Therefore, technical and infrastructure capacity for malaria control should be strengthened to facilitate implementation, surveillance, monitoring, and evaluation.

## Abbreviations

ACT: artemisinin-based combination therapy
BCC: behaviour change communication
EA: enumeration area
ECOSOC: economic and social consulting
IEC: information, education and communication
IPT: intermittent preventive treatment
IRS: indoor residual spraying
ITNs: insecticide-treated nets
IVM: integrated vector management
LLINs: Long-lasting insecticidal nets
LSM: larval source management
NHL: National Health Laboratory
NSO: National Statistics Office
MERG: Monitoring and Evaluation Reference Group
MIS: malaria indicator survey
MoH: Ministry of Health
NMCP: National Malaria Control Programme
SP: sulfadoxine-pyrimethamine
RBM: roll back malaria
RDTs: rapid diagnostic tests
WHO: World Health Organization
